# Endovascular revascularisation in chronic occlusive mesenteric ischaemia: safety and efficacy of intravascular lithotripsy

**DOI:** 10.1007/s00330-025-12310-9

**Published:** 2026-01-30

**Authors:** Annette Thurner, Dominik Peter, Sven Lichthardt, Anne Marie Augustin, Sven Flemming, Ralph Kickuth

**Affiliations:** 1 Department of Diagnostic and Interventional Radiology, University Hospital Würzburg, Oberdürrbacher Str. 6, 97080 Würzburg, Germany; 2 Department of General, Visceral, Transplantation, Vascular and Paediatric Surgery, University Hospital Würzburg, Oberdürrbacher Str. 6, 97080 Würzburg, Germany; 3 Department of Radiology, Neuroradiology and Interventional Therapy, Klinikum Bayreuth GmbH, Preuschwitzer Str. 101, 95445 Bayreuth, Germany

**Keywords:** Intravascular lithotripsy, Chronic occlusive mesenteric ischaemia, Calcium modification, Mesenteric artery stenting

## Abstract

**Objective:**

To evaluate the safety and efficacy of intravascular lithotripsy (IVL)-assisted endovascular revascularisation in patients with chronic mesenteric ischaemia (CMI) and heavily calcified mesenteric artery stenoses.

**Materials and methods:**

In this single-centre retrospective study (May 2020–June 2025), consecutive patients with symptomatic CMI, ≥ 50% mesenteric artery stenosis, and moderate-to-severe calcification on CT angiography underwent IVL-assisted endovascular revascularisation. Outcomes included technical success (successful IVL with ≤ 30% residual stenosis after any adjunctive therapy), moderate-to-severe adverse events (AEs), symptom recurrence, clinically driven target vessel revascularisation (CD-TVR), patency, and survival. Kaplan-Meier analysis assessed patency and survival at 6 and 12 months.

**Results:**

Fifty-one patients (median age, 71.5 years; 51% women) underwent treatment of 57 arteries (median stenosis, 72.0%; 96.5% moderate-to-severe calcification). IVL was followed by stenting in 53 de-novo lesions (47 bare-metal, 6 covered), and balloon angioplasty in 4 lesions (3 de-novo, 1 in-stent restenosis). Technical success was 93.0%, with predilatation required in 45.6% of vessels. Median residual stenosis was 16.7% (IQR 11.7), and median lumen gain was 3.5 mm (IQR 2.1). Moderate-to-severe AEs occurred in 27.5% of patients. Two patients were lost to follow-up. During a median follow-up of 578.0 days (IQR 529.5), symptom recurrence occurred in 18.4% of patients, and CD-TVR was required in 16.3%. Primary clinical patency was 93.4% at 6 months and 91.0% at 12 months. Survival rates were 91.7% and 89.4% at 6 and 12 months, respectively; mesenteric ischaemia-related mortality was 2.0%.

**Conclusion:**

IVL is a safe and effective vessel preparation strategy for heavily calcified mesenteric arteries, facilitating endovascular revascularisation in CMI.

**Key Points:**

***Question*** *Can vessel preparation with intravascular lithotripsy reduce the rate of endovascular treatment failure associated with moderate-to-severe calcification in mesenteric artery stenosis without amplifying procedural risks?*

***Findings**** Calcium modification with intravascular lithotripsy prior to stenting yielded high technical and clinical success with favourable lumen gain, safety profile, and durable patency*.

***Clinical relevance**** Adjunctive intravascular lithotripsy is a valuable strategy to mitigate the challenges of calcification in mesenteric artery stenosis, achieving high technical and clinical success while preserving procedural safety, thereby broadening treatment feasibility and improving outcomes in complex disease*.

**Graphical Abstract:**

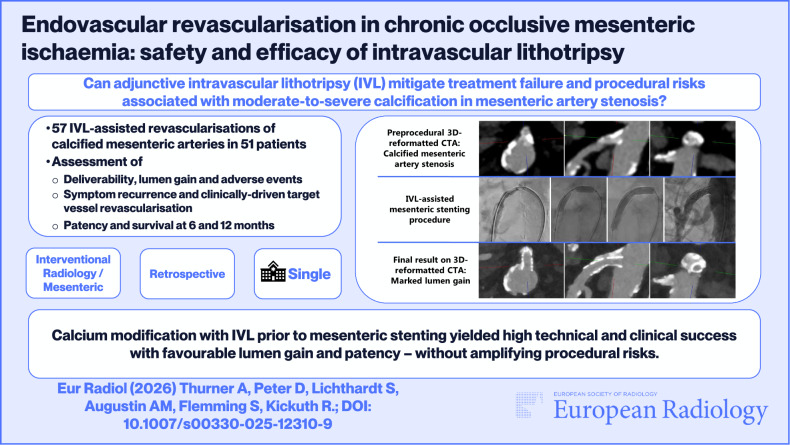

## Introduction

Endovascular revascularisation is the preferred treatment for atherosclerotic chronic occlusive mesenteric ischaemia (CMI), particularly in elderly and comorbid patients, due to its lower invasiveness and periprocedural risk compared with open mesenteric bypass [1, 2]. However, endovascular therapy is associated with higher rates of early restenosis, symptom recurrence, and re-intervention, underscoring the need for procedural optimisation [1, 3].

A major factor limiting endovascular treatment success is high calcium burden, which is common in mesenteric artery stenosis. Calcifications limit procedural success by impeding lesion crossability, reducing predilatation efficacy, compromising stent expansion, and increasing risk of dissection and perforation [4–6]. Moderate-to-severe calcification and residual stenosis are well-established predictors of in-stent restenosis (ISR), thrombosis, and symptom recurrence [5, 6]. Therefore, effective measures to optimise lumen gain while maintaining procedural safety are essential to improve outcomes in endovascular treatment of CMI.

Conventional vessel preparation and plaque modification tools often perform suboptimally in this vascular territory. High-pressure, cutting, or scoring balloons can cause asymmetric expansion, particularly along the fibrocalcific interface, predisposing vessels to dissection or perforation [5, 7, 8]. Atherectomy is rarely used in mesenteric arteries due to vessel tortuosity, eccentric wire bias, and the heightened risk of perforation or distal embolisation [5, 7, 8].

Intravascular lithotripsy (IVL) represents a balloon-based calcium modification technology that combines balloon angioplasty and calcified plaque disruption through the delivery of pulsatile sonic pressure waves, analogous to lithotripsy for renal calculi [9, 10]. Electrohydraulic emitters integrated within a single-use angioplasty balloon generate spherical outward-radiating acoustic pressure waves that selectively fracture both intimal and medial calcium, thereby increasing vessel compliance and facilitating optimal angioplasty or stent expansion [5, 9, 10]. IVL has demonstrated improved vessel compliance, lumen gain, and stent expansion with low complication rates in coronary and peripheral arteries, suggesting also potential benefit in CMI [5, 11–13].

While early reports and small series point to promising outcomes with IVL-assisted mesenteric artery stenting in terms of acute safety and effectiveness, evidence remains limited [13]. The authors have previously demonstrated the feasibility of IVL in mesenteric artery revascularisation for CMI [14]. The present study aims to evaluate technical success, safety, patency, and survival in a larger cohort with extended follow-up.

## Materials and methods

### Patients and study design

This retrospective single-centre observational study included all patients who underwent endovascular revascularisation of mesenteric arteries at a tertiary referral centre between May 2020 and June 2025. From this cohort of 107 patients, 51 consecutively selected adults treated with IVL-assisted revascularisation for CMI were identified and included in the analysis. The study was approved by the institutional ethics committee (2025-294-dvhd), and written informed consent was provided by the patients at least 24 h before the procedure. The study was conducted in accordance with the Declaration of Helsinki and followed the STROBE reporting guidelines.

Eligible patients presented with abdominal angina and/or endoscopically confirmed ischaemic gastritis/colitis, together with computed tomography angiography (CTA) evidence of relevant mesenteric artery stenosis or chronic total occlusion (CTO) [1, 2, 15]. In symptomatic patients, relevant stenosis was defined as ≥ 70% in single-vessel disease, and ≥ 50% in multi-vessel disease [15]. In addition, all included lesions demonstrated moderate-to-severe calcification according to the Society of Vascular Surgery classification [16], or were non-dilatable by percutaneous transluminal angioplasty (PTA) with a residual stenosis ≥ 50% [5, 7]. Other eligible morphologies included calcific segments exceeding 15 mm, negatively remodelled calcified vessels, and a calcified nodule protruding into the lumen [5, 7]. IVL was also considered in cases of stent underexpansion due to calcium, calcific in-stent neo-atherosclerosis, or calcified nodular re-protrusion [5, 17, 18].

**Fig. 1:**
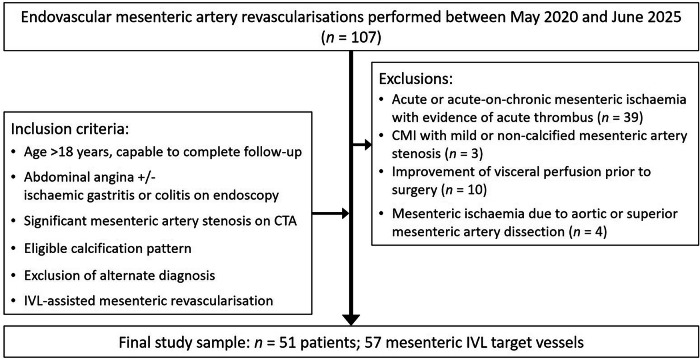
Flowchart illustrating patient selection. CMI, chronic mesenteric ischaemia; CTA, computed tomography angiography; IVL, intravascular lithotripsy

Patients with acute or acute-on-chronic occlusive mesenteric ischaemia accompanied by acute thrombus, non-calcified mesenteric artery stenosis, or those undergoing mesenteric artery stenting to optimise visceral perfusion before gastrointestinal or pancreatic surgery, were excluded. Additional exclusion criteria were dissection, vasculitis, and coeliac artery (CA) stenosis due to median arcuate ligament compression [2, 15]. The patient selection process is illustrated in Fig. 1.

### Intravascular lithotripsy device and procedure

All procedures were performed under local anaesthesia in a dedicated angiography suite (Azurion Clarity IQ, Philips) by a board-certified interventional radiologist with 27 years of experience. Femoral access was preferred. Intra-procedural anticoagulation was achieved with heparin (80–100 U/kg).

Selective digital subtraction angiography of the mesenteric arteries was performed using 5-Fr cobra- or sidewinder-shaped diagnostic catheters. The diagnostic sheath was then exchanged for a 6- or 7-Fr guiding sheath (Destination, Terumo). After lesion crossing with a 0.014-inch guidewire (J Tip CHOICE PT Extra Support, Boston Scientific), IVL was performed using peripheral or coronary IVL balloons sized 1:1 to the reference vessel diameter (Shockwave M5, M5+, S4 or C2+, Shockwave Medical; diameters 4.0–7.0 mm, lengths 12–60 mm) [5, 8, 9].

The IVL balloon was inflated to 4 atm to achieve balloon-vessel wall apposition and connected to a portable generator delivering pulsatile sonic pressure waves. These acoustic waves propagate circumferentially through soft tissue and selectively fracture intimal and medial calcium without removing plaque material. The effective penetration depth of IVL energy varies by balloon design and has been reported to reach approximately 3–7 mm [11]. Following calcium modification, the balloon was further inflated to 6 atm to optimise lumen gain, with the sequence of calcium cracking and angioplasty repeated until satisfactory expansion was achieved.

For peripheral IVL catheters, a minimum of 150-180 pulses per lesion (in 30-pulse cycles) was delivered, up to a device limit of 300 pulses. Coronary IVL catheters were limited to 120 pulses per lesion. Overall, a median of 194.4 (IQR 20) pulses per lesion was applied.

When IVL balloon advancement was unsuccessful, predilatation with semicompliant low-profile balloons (0.014−/0.018-inch delivery platforms, diameters 2.5–5 mm, lengths 20–40 mm; Armada 14/18, Abbott, or Pacific Plus, Medtronic) was performed to facilitate crossing.

Adjunctive technologies were employed at the operator’s discretion, including balloon angioplasty and/or mesenteric artery stenting. Stenting was performed using standard techniques, primarily with bare-metal stents (BMS; 88.7%) and, in selected cases, covered stents (CSs; 11.3%), sized 4–10 mm in diameter and 12-38 mm in length. Most stents were 0.035-inch cobalt-chromium BMS (71.7%), while low-profile stainless steel or cobalt-chromium BMS designs accounted for 17%. Six cobalt-chromium CSs were used. No drug-eluting stents were deployed. Supplementary Material presents detailed technical specifications of commonly selected materials and devices.

Intraprocedural adverse events (AEs) were managed according to standard practice, including aspiration thrombectomy for distal embolisation. No embolic protection devices were used during the procedure. Guide-support techniques, including sheath-support, were employed for both IVL and stenting as required. The choice of access site, stent design, and other procedural materials was left to the operator’s discretion.

For patients with more than one eligible calcified stenotic mesenteric artery requiring treatment, IVL was applied to several mesenteric vessels. Any additional stenotic mesenteric arteries that were not eligible for IVL but required intervention were treated as non-target lesions without IVL during the same session. Additional non-target lesions were treated in 21.6% (11/51) of patients.

### Follow-up and data collection

All patients underwent clinical surveillance for at least 24 h after the procedure. After discharge, follow-up visits were scheduled at 3, 6 and 12 months in accordance with institutional standards. Clinical assessment was supplemented by duplex ultrasound and/or CTA, taking into account patient comorbidities and renal function.

Post-procedural antithrombotic strategies were tailored to the patient’s comorbidity profile, thromboembolic risk, and drug tolerability. In general, dual antiplatelet therapy was recommended for 6 months after endovascular revascularisation, followed by lifelong single antiplatelet therapy [1, 2, 19]. For patients with an independent indication for anticoagulation (e.g., atrial fibrillation), combination therapy was minimised with a single antiplatelet agent restricted to the first four weeks after revascularisation to mitigate bleeding risk.

Medical and imaging records were analysed retrospectively to obtain information on demographics, comorbidities, mesenteric vascular status, procedural characteristics, and follow-up outcomes.

### Endpoints and definitions

The following outcomes were assessed: technical success, safety, symptom recurrence, clinically driven target vessel revascularisation (TVR), patency, and survival. Technical success was defined as successful IVL delivery to the target lesion with ≤ 30% residual diameter stenosis on final angiography. The safety outcome was defined as the absence of both moderate-to-severe procedural adverse events (AEs), including flow-limiting dissection (National Heart, Lung, and Blood Institute (NHLBI) types C-F), vessel perforation, acute closure, distal embolisation, reperfusion injury, and AEs at the access site requiring intervention. AEs were graded according to the Society of Interventional Radiology [20]. Primary clinical patency was defined as freedom from recurrent symptoms of mesenteric ischaemia, with no requirement for endovascular clinically-driven target vessel revascularisation (CD-TVR). Primary assisted clinical patency was defined as clinical patency following endovascular CD-TVR, and secondary clinical patency as clinical patency following surgical bypass. Patency assessment was primarily symptom-based due to imaging limitations in calcified, stented vessels; routine invasive angiography was not performed in asymptomatic patients.

### Statistical analysis

Categorical variables were expressed as counts and percentages, and continuous variables as mean ± standard deviation for normally distributed data or median with interquartile range (IQR) for non-normally distributed data. Normality was assessed using the D’Agostino-Pearson test. Between-group comparisons were performed using the Mann-Whitney test for two unpaired groups or the Kruskal-Wallis test for more than two unpaired groups, as appropriate. Kaplan-Meier analysis was used to estimate primary clinical patency and survival at 6 and 12 months. A sensitivity analysis was conducted to evaluate the potential impact of missing data for the outcome, which were handled using three different strategies: (1) imputing as “patency lost/death,” (2) imputing as “patency maintained/alive,” and (3) excluding incomplete data sets. The variability in estimated rates across these methods was modest (approximately 4%), which was deemed acceptable given the cohort’s limited size. Accordingly, the two datasets with incomplete data for outcome assessment were excluded. A *p*-value less than 0.05 was considered statistically significant. Statistical analyses were performed using GraphPad Prism (GraphPad Software).

## Results

### Patient characteristics and mesenteric vascular status

Fifty-one patients with CMI and calcified mesenteric artery stenosis underwent 57 IVL-assisted target vessel revascularisations and were eligible for retrospective analysis. The patients’ median age at presentation was 71.5 years (IQR 12), and 51.0% (26/51) were females. Two patients were lost to follow-up. Median follow-up was 578.0 days (IQR 529.5) with a 12-month follow-up compliance of 91.8% (45/49).

At presentation, 90.2% (46/51) of patients reported postprandial pain and food fear, and 78.4% (40/51) had unintended weight loss (median 3.1 kg/month (IQR 3.75)) over 1-24 months. Endoscopically confirmed ischaemic gastritis/colitis was documented in 51.0% (26/51).

Single-vessel disease, affecting one of the three mesenteric arteries, was present in 7.8% (4/51), double-vessel disease in 29.4% (15/51), and triple-vessel disease in 62.8% (32/51). Median lesion length was 11.4 mm (IQR 9), with 28.1% (16/57) exceeding 15 mm. Median baseline diameter stenosis was 72.0% (IQR 23.3). CTOs accounted for 8.8% (5/57). Ostial involvement predominated. Plaque morphology was mixed in 52.6% (30/57) and calcified in 47.4% (27/57); 21.1% (12/57) were moderately calcified, and 75.4% (43/57) were severely calcified. Two cases with mild calcification—one non-dilatable CTO and one chronic non-dilatable ISR—were treated with IVL due to ≥ 50% residual stenosis after PTA. Patient characteristics and mesenteric artery status are provided in Table 1.

**Table 1:** Patient characteristics and mesenteric artery status

Parameters	Value (*n* = 51 patients; 57 target vessels)
Age, years	71.5 (IQR 12)
Sex (female/male)	26/25 (51.0/49.0)
Body mass index, kg/m²	25.7 (IQR 6.92)
**Risk factors**	
Hypertension	47 (92.2)
Diabetes mellitus	24 (47.1)
Dyslipidaemia	38 (74.5)
Current/heavy smokers (> 20 pack-years)	13 (25.5)
Coronary artery disease/Coronary bypass surgery/Cardiac insufficiency/Ischaemic cardiomyopathy/Cardiac arrhythmia	26 (51.0)
Peripheral artery disease/Aortic aneurysm	30 (58.8)
Chronic kidney disease, stage 1-2 (GFR > 60 mL/min/1.73 m^2^)	30 (58.8)
Chronic kidney disease, stage 3 (GFR 30–59 mL/min/1.73 m^2^)	16 (31.4)
Chronic kidney disease, stage 4-5 (GFR ≤ 29 mL/min/1.73 m^2^ or dialysis)	5 (9.8)
Chronic obstructive pulmonary disease	7 (13.7)
Stroke/Carotid surgery	9 (17.6)
Liver cirrhosis	1 (2.0)
Anaemia (haemoglobin < 13.5 g/dl; haematocrit < 40%)	38 (74.5)
Any antithrombotic therapy prior to intervention	42 (82.4)
Coagulopathy (Factor-V-Leiden; acquired hembody haemophilia)	2 (3.9)
Malignancy	14 (27.5)
Prior bowel resection due to ischaemia	2 (3.9)
**Clinical signs and symptoms**	
Postprandial pain/food fear	46 (90.2)
Unintended weight loss	40 (78.4)
Weight loss per month, kg/month	3.1 (3.75)
Ischaemic gastritis/colitis	26 (51.0)
(Bloody) diarrhoea	23 (45.1)
**Mesenteric artery disease distribution**	
Single vessel disease	4 (7.8)
Double vessel disease	15 (29.4)
Triple vessel disease	32 (62.8)
**Calcification severity**	
Mild (< 25% circumference)	2 (3.5)
Moderate (25%–50%)	12 (21.1)
Severe (> 50%)	43 (75.4)
**Baseline target lesion characteristics**	
Reference vessel diameter, mm	6.2 (IQR 1.6)
Minimum lumen diameter, mm	1.7 (IQR 1.5)
Baseline diameter stenosis, %	72.0 (IQR 23.3)
Lesion length, mm	11.4 (IQR 9)
Chronic total occlusions	5 (8.8)

Data are presented as *n* (%), *n*/*N* (%), or median (interquartile range, IQR)

*GFR* glomerular filtration rate

### Procedure characteristics and technical success

IVL delivery was successful in all lesions; additional predilatation was required in 45.6% (26/57). IVL balloon rupture occurred in 3.5% (2/57). A single target vessel was addressed in 88.2% (45/51) of patients, while 11.8% (6/51) underwent double-vessel treatment. The IVL target vessels included 38 superior mesenteric arteries (SMA; 66.6%), 12 coeliac arteries (21.1%), 4 inferior mesenteric arteries (IMA; 7%), and 3 celiacomesenteric trunks (5.3%). Median acute lumen gain after IVL was 1.6 mm (IQR 1.3) while median residual diameter stenosis was 43.9% (IQR 13.8).

Post-IVL PTA was performed in 17.5% (10/57), and 93.0% (53/57) of target lesions were stented. For PTA after IVL, eight plain balloons and two drug-eluting balloons were used. The two drug-eluting balloons were employed in one de-novo lesion of a celiacomesenteric trunk and in the management of a non-dilatable ISR of the SMA. Early in the study, three de-novo lesions (two SMA, one IMA) were treated with IVL and PTA alone, achieving low residual stenosis; however, both SMA cases required secondary stenting due to symptom recurrence at 3 and 9 months, respectively. The IMA patient was lost to follow-up.

A total of 53 balloon-expandable stents were deployed (4 overlapping constructs). Minor stent migration occurred in two overlapping deployments, necessitating extended coverage; no stent delivery failure or stent luxation occurred.

**Fig. 2:**
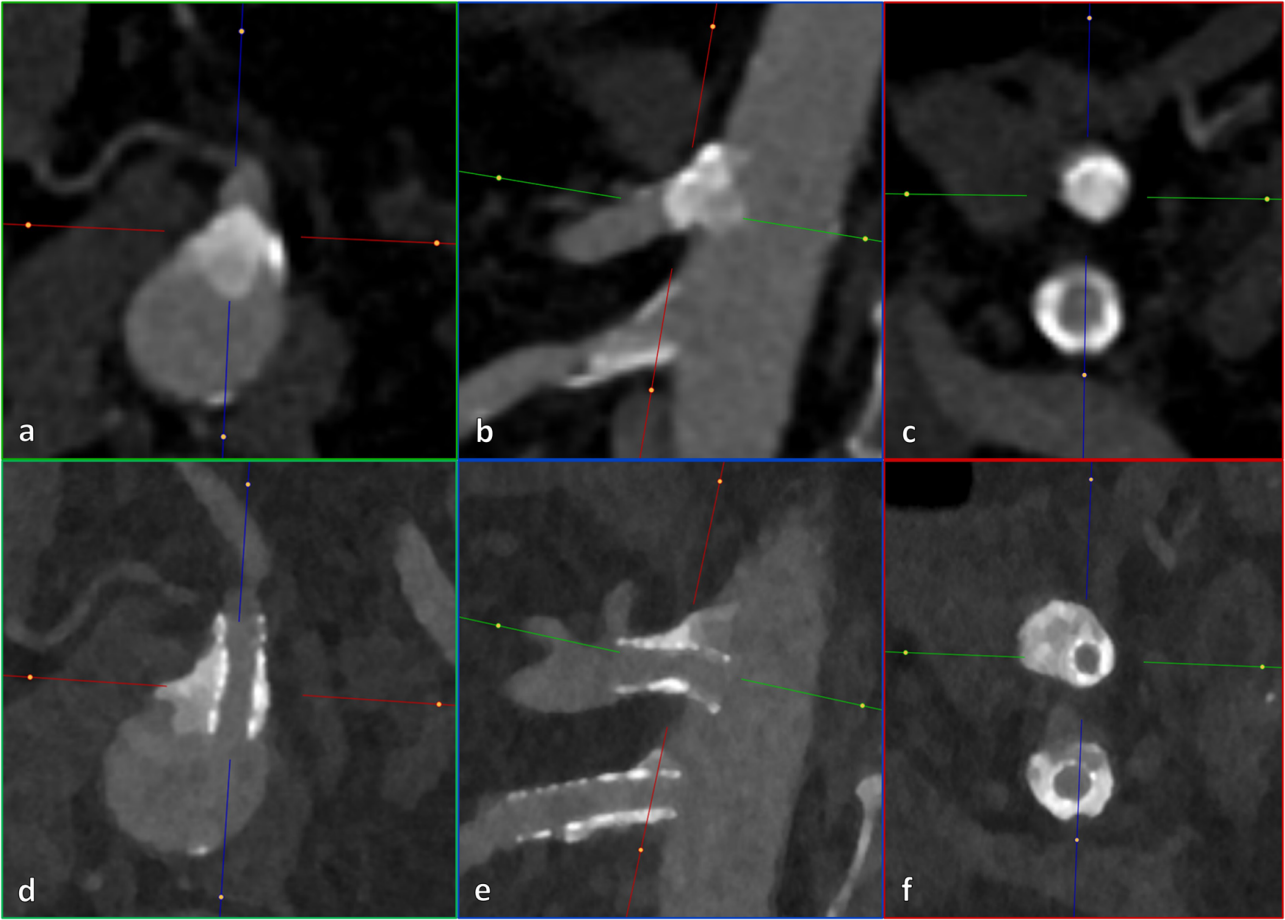
3D-reformatted CT angiography in paraaxial (green), parasagittal (blue), and paracoronal (red) views before and after IVL-assisted double-vessel bare-metal stent placement in a 64-year-old patient with chronic mesenteric ischaemia. The upper row (**a**–**c**) shows the preprocedural status of the superior mesenteric artery and coeliac artery: a calcified coeliac short-segment chronic total occlusion, and a relevant superior mesenteric artery stenosis due to a mixed atherosclerotic plaque with circumferential calcium and negative remodelling. The lower row (**d**–**f**) demonstrates postprocedural results with substantial lumen gain, favourable stent expansion, and only mild residual stenosis at the point of maximal calcification

Median residual stenosis at final angiography was 16.7% (IQR 11.7), representing a median stenosis reduction of 76.0% (IQR 18.2) and a median acute lumen gain of 3.5 mm (IQR 2.1). Figure 2 shows CTA images before and after IVL-assisted double-vessel stent placement. Technical success was achieved in 93.0% (53/57). Residual stenosis > 30% occurred in one SMA (33% residual stenosis), one CA (38% residual stenosis) and two IMAs (55% and 66% residual stenoses). Low-profile stents were associated with significantly higher levels of residual stenosis than standard profile stents (*p* = 0.03). Table 2 details procedure characteristics.

**Table 2:** Procedure characteristics

Parameters	Value (*n* = 51 patients; 57 target vessels)
**Access route**	
Femoral	50 (98.0)
Brachial	1 (2.0)
**Intravascular lithotripsy**	
IVL target vessels	
Superior mesenteric artery	38 (66.6)
Coeliac artery	12 (21.1)
Inferior mesenteric artery	4 (7.0)
Celiacomesenteric trunk	3 (5.3)
Number of target vessels treated per procedure	
Single	45 (88.2)
Double	6 (11.8)
Successful IVL delivery across the target lesion	57 (100)
Pulses per lesion	194.4 (IQR 20)
IVL balloon rupture	2 (3.5)
**Adjunctive therapy**	
Pre-dilatation	26 (45.6)
Post-IVL dilatation (8× POBA, 2× DEB-PTA)	10 (17.5)
Bare-metal stent	47 (88.7)
0.014/0.018-inch platform (*Tsunami*, Terumo; *RX Herculink Elite Peripheral*, Abbott; *Palmaz Blue*, Cordis)	9 (17.0)
0.035-inch platform (*BeSmooth*, Bentley InnoMed; *Visi-Pro*, Medtronic)	38 (71.7)
Covered stent	6 (11.3)
0.014-inch platform (*PK Papyrus*, Biotronik)	1 (1.9)
0.035-inch platform (*BeGraft*, Bentley InnoMed)	5 (9.4)
**Final result**	
Acute lumen gain, mm	3.5 (IQR 2.1)
Minimum lumen diameter, mm	5.3 (IQR 1.9)
Residual diameter stenosis, %	16.7 (IQR 11.7)
< 30%/> 50%	55/2 (96.5/3.5)
Relative stenosis reduction	76.0 (IQR 18.2)
**Procedural adverse events**	
Intra-procedural	
Mild AEs	
Self-limiting vasospasm	3 (5.9)
Non-flow-limiting dissection (NHLBI A/B)	2 (3.9)
Moderate-to-severe AEs	
Flow-limiting dissection (NHLBI D)	3 (5.9)
Distal embolisation	2 (3.9)
Reperfusion haemorrhage gastroduodenal artery	1 (2.0)
Cardiac decompensation requiring intensive care	1 (2.0)
Early post-procedural period (POD 1-3)	
Access site complication requiring intervention (POD 1)	1 (2.0)
Acute stent thrombosis (POD 1)	1 (2.0)
Death on POD 1 due to myocardial infarction	1 (2.0)
Mild reperfusion injury (POD 2-3)	3 (5.9)
Perforated cholecystitis with peritonitis (POD 3)	1 (2.0)

Data are presented as *n* (%), *n*/*N* (%), or median (interquartile range, IQR)

*AEs* adverse events, *IVL* intravascular lithotripsy, *POBA* plain old balloon angioplasty, *DEB-PTA* drug-eluting balloon angioplasty, *POD* post-procedural day, *NHLBI* National Heart, Lung, and Blood Institute

### Safety

The overall procedural AE rate was 37.3% (19/51), with 27.5% (14/51) classified as moderate-to-severe.

Intraprocedural mild AEs occurred in 9.8% (5/51), including self-limiting vasospasms and non-flow-limiting dissections; intraprocedural moderate-to-severe AEs occurred in 13.7% (7/51): distal embolisation in two CTOs (treated with aspiration thrombectomy), one gastroduodenal artery reperfusion haemorrhage (excluded with CS), three flow-limiting dissections (treated with stenting), and one cardiac decompensation (managed with intensive care). No target vessel perforation or acute closure occurred.

During post-procedural days (POD) 1–3, seven further moderate-to-severe AEs (13.7%) were observed: one acute limb ischaemia from femoral artery dissection/thrombosis (surgical repair), one acute stent thrombosis (endovascular treatment), one perforated cholecystitis with peritonitis (treated surgically), three transient reperfusion injuries (managed conservatively), and one fatal myocardial infarction.

The three reperfusion injuries presented with abdominal pain, malaise, and elevated C-reactive protein, while lactate, liver enzymes, and lipase remained normal. Imaging excluded target vessel occlusion or embolism; one case showed oedematous/inflammatory changes of the right hemicolon. It remains unclear whether cardiac decompensation and cholecystitis were due to reperfusion injury or independent events.

### Patency

Symptom recurrence occurred in 9/49 patients (18.4%), with 8 (16.3%) requiring CD-TVR during follow-up. CD-TVR rates were 6.1% (3/49) at 6 months, 10.2% (5/49) at 12 months, and 16.3% (8/49) at 24 months. One patient developed acute liver injury from thrombotic coeliac stent occlusion; conservative management restored liver function without recurrent CMI symptoms or further ischaemic events.

**Fig. 3:**
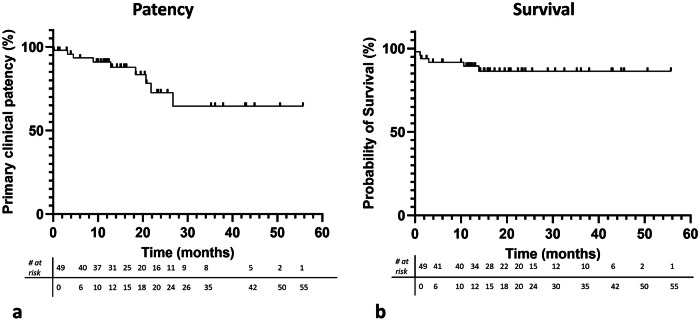
Kaplan–Meier curves of primary clinical patency and survival. The estimated primary clinical patency rate after 6 and 12 months was 93.4% and 91.0% (**a**). The estimated survival rate after 6 and 12 months was 91.7% and 89.4% (**b**)

Kaplan-Meier primary clinical patency was 93.4% at 6 months and 91.0% at 12 months (Fig. 3a), with no significant differences between BMS and CS (*p* = 0.25) or the underlying mesenteric artery disease distribution (*p* = 0.27). Median primary assisted clinical patency after CD-TVR was 299.8 days (IQR 383.5). Three patients required bypass surgery for symptomatic re-occlusion after endovascular CD-TVR, yielding a median secondary clinical patency of 185.0 days (IQR 120); one bypass required revision, and one occluded asymptomatically.

Asymptomatic mild ISR (< 50%) occurred in 5/49 (10.2%) and was managed conservatively. One patient developed recurrent moderate ISR in the SMA after IVL and drug-eluting balloon angioplasty but remained asymptomatic for 55 months under conservative treatment up to census. Two cases of stent compression from calcified nodular re-protrusion with moderate late lumen loss were identified on CTA without the need for re-intervention.

### Survival

During follow-up, 16.3% (8/49) of patients died. Causes included acute-on-chronic mesenteric ischaemia (CMI) (*n* = 1), cardiopulmonary disease (*n* = 4), sepsis (*n* = 1), malignancy (*n* = 1), and unknown (*n* = 1). Mesenteric ischaemia-related mortality was 2.0% (1/49). Kaplan–Meier survival estimates were 91.7% at 6 months and 89.4% at 12 months (Fig. 3b).

## Discussion

The present study findings indicate that vessel preparation with IVL, which combines balloon angioplasty with pulsatile sonic pressure waves to fragment intimal and medial calcified plaque, effectively facilitates stent implantation in calcified mesenteric artery stenosis, achieving high technical success, substantial lumen gain, minimal residual stenosis, and acceptable rates of AEs across heterogeneous calcification patterns.

The overall procedural AE rate of 37.3%, with 27.5% clinically relevant moderate-to-severe AEs, is consistent with prior reports and suggests that IVL does not confer additional procedural risks [5, 11, 19]. For example, a multicentre randomised controlled trial of non-IVL-assisted CMI treatment using CS and BMS reported overall procedural AE rates between 19% and 36% [19].

Despite these encouraging results, IVL in visceral arteries presents specific technical challenges, particularly in device stability and deliverability. In this series, predilatation was required in 45.6% of cases, a rate higher than that reported in IVL studies on lower-limb peripheral artery disease (31%) but slightly lower than in coronary IVL (55.2%) [10, 12]. Difficulties arose from complex calcifications and tortuous transfemoral or long transbrachial access routes, complicating lesion crossing over a 0.014-inch guidewire. Nevertheless, a successful IVL application was achieved in all cases using predilatation and guide support techniques.

With regard to vessel wall injury, the dissection rates observed in this study (5.3% for types D-F, 3.5% for types A-C) were at the upper end of those previously reported. In coronary IVL, Hill et al reported a 0.3% rate of flow-limiting dissections [10], whereas Ali et al observed a 13% rate [9]. Lower-limb IVL studies found type D-F dissection rates of 0% to 1.4% [21, 22]. For non-IVL-assisted mesenteric stenting, Terlouw et al reported a 2% rate [19], and Oderich et al documented 1.3% [23]. Although the sample size is insufficient to identify definitive risk factors, the combination of dissection events and IVL balloon ruptures warrants reconsideration of the current practice of inflating IVL balloons to 6 atm after each pulse set – a technique no longer recommended in calcified coronary lesions due to its association with increased balloon rupture and, possibly, dissections [5].

IVL was applied in five CTOs, with two cases of distal embolism requiring aspiration thrombectomy. CTOs present specific challenges in vessel preparation, particularly regarding the uncertainty of intra- versus extraplaque crossing. Current recommendations favour IVL in CTOs where subintimal routing is suspected, and calcium modification is required [5]. However, the unpredictable presence of appositional thrombus in mixed plaque morphologies increases the risk of thrombus detachment and embolisation. In such cases, non-IVL-assisted stenting with CSs may be a reasonable option.

In contrast to other vascular territories [21], IVL in mesenteric arteries should be considered as a vessel preparation technique before stenting, rather than a stand-alone modality. This is supported by the symptom recurrences following IVL and PTA alone in this study.

The calcification pattern of IMA stenosis is another limitation for IVL. These lesions often arise from long-segment, circumferential aortic wall calcification, resulting in ultra-short ostial IMA stenosis with insufficient IVL balloon-calcium contact and suboptimal transmission of lithotripsy waves [24, 25]. Consequently, residual stenosis ≥ 50% was observed in two IMA cases.

In contrast to Terlouw et al, who reported a 6% luxation rate for CS (0.035-inch platforms) [19], no cases of stent luxation occurred in this study. The systematic use of upfront IVL and sheath-supported/protected stent delivery may explain this finding, as both strategies facilitate safe passage across tortuous segments and heavily calcified ostial lesions.

The data also suggest a potential role for IVL in improving patency and reducing the need for re-intervention. Favourable CD-TVR rates were observed at 6 (6.1%) and 12 (10.2%) months, with primary clinical patency rates of 93.4% and 91.0%, respectively. These outcomes compare more favourably with previous non-IVL-assisted BMS series (low-profile stents) [19], which demonstrated higher re-intervention rates (8% at 6 months and 26% at 12 months) and lower primary patency rates (80% at 6 months and 62% at 12 months) [19]. Similarly, Haben et al demonstrated 12-month primary patency rates of 80.5% in the SMA and 85.9% in the CA following non-IVL-assisted BMS use [26].

The study findings also align with the excellent outcomes reported for non-IVL-assisted CS implantation (0.035-inch delivery platforms) [19, 27]. Terlouw et al reported similar re-intervention rates (5% at 6 months and 11% at 12 months) and primary patency rates (92% at 6 months and 90% at 12 months) [19]. Nevertheless, the relative trade-offs between BMS and CS remain clinically relevant. While CS provides durable patency, its use has been associated with higher rates of technical failure and target-vessel thrombosis [19]. In contrast, BMS are more prone to ISR [19], but this complication is typically easier and safer to manage than acute thrombosis.

Finally, following IVL-assisted stenting, standard-profile stents (0.035-inch delivery platforms) provided superior lumen gain compared with low-profile stents (0.018-inch delivery platforms), underscoring the need for further investigation.

Several limitations have to be acknowledged with regard to the study design. This study was not designed to provide a direct comparison between IVL-assisted BMS and CS implantation using equivalent platforms, and stent selection remains a key determinant of outcomes in calcified lesions. Larger prospective multi-centre trials with standardised stent platforms and extended follow-up are needed to define the role of IVL in mesenteric revascularisation. Potential trial designs include comparisons of (1) standard bare-metal stenting (BMS) versus IVL-assisted BMS, and (2) IVL-assisted BMS versus IVL-assisted CS implantation. In the authors’ opinion, future investigations should ensure the use of comparable stent platforms between treatment arms (BMS and CS) and incorporate 0.035-inch delivery systems. Guiding sheath-protected stent delivery should be considered mandatory to standardise device support, optimise lesion access, and minimise deployment variability. Additional factors, including collateralisation, stent flaring, and periprocedural pharmacotherapy (e.g., statins, anticoagulants), may also affect long-term patency and require further evaluation [26, 28]. Although the missing data of the two patients lost to follow-up introduces bias, the sensitivity analysis demonstrated that the effect of the missing data on the calculations of primary patency and survival rates was limited. Finally, the retrospective design and absence of a randomised control group limit the strength of causal inferences.

In conclusion, IVL is a safe and effective vessel preparation strategy for stenting in calcified mesenteric artery stenosis, achieving high technical success with acceptable AE rates.

## Supplementary information

Supplementary Material

## Abbreviations

AE: Adverse event
Atm: Atmosphere
BMS: Bare-metal stent
CA: Coeliac artery
CD-TVR: Clinically driven target vessel revascularisation
CMI: Chronic mesenteric ischaemia
CS: Covered stent
CTA: Computed tomography angiography
CTO: Chronic total occlusion
Fr: French
IMA: Inferior mesenteric artery
IQR: Interquartile range
ISR: In-stent restenosis
IVL: Intravascular lithotripsy
NHLBI: National Heart, Lung, and Blood Institute
POD: Post-procedural day
PTA: Percutaneous transluminal angioplasty
SMA: Superior mesenteric artery
