# Supplementary material for: Endovascular revascularisation in chronic occlusive mesenteric ischaemia: safety and efficacy of intravascular lithotripsy
Source: Eur Radiol. 2026 Jan 30;36(6):4658–68. doi: 10.1007/s00330-025-12310-9 (PMC13212681; doi:10.1007/s00330-025-12310-9)
Supplement: Supplementary file 1 — Supplementary Material [file 330_2025_12310_MOESM1_ESM.pdf]

Endovascular Revascularisation in Chronic Occlusive Mesenteric Ischaemia: Safety and Efficacy of Intravascular Lithotripsy

ELECTRONIC SUPPLEMENTARY MATERIAL

-

1) Patient Characteristics and Mesenteric Vascular Status

2) Procedure Characteristics, IVL Performance, Adjunctive Therapy, Safety Outcomes, and Follow-up

3) Technical Specifications of Commonly used Materials and Devices

4) Sensitivity Analysis – Missing Follow-up Data Handling

5) Box-Whisker Plots: Diameter Stenosis and Minimal Lumen Diameter at Baseline, after IVL and on the Final Angiogram

1) Patient Characteristics and Mesenteric Vascular Status	
**Parameter/Outcome**	**Value**
Demographics	
Age, years	71.5 (12; 52-89)
Male sex	25 (49.0)
Female sex	26 (51.0)
Body weight, kg	72.1 (23; 38-146)
Body mass index, kg/m²	25.7 (6.9; 16.23-40.0)
Medication prior to intervention	
Any antithrombotic therapy	42 (82.4)
Single antiplatelet therapy (aspirin, clopidogrel, or ticagrelor)	25 (49.0)
Dual antiplatelet therapy	5 (9.8)
Anticoagulant – direct factor Xa inhibitor	6 (11.8)
Anticoagulant – direct thrombin inhibitor	0 (0)
Anticoagulant – vitamin K antagonist	2 (3.9)
Combination therapy (antiplatelet + anticoagulant)	4 (7.8)
Statin therapy (HMG-CoA reductase inhibitor)	38 (74.5)
Risk factors and comorbidities	
Hypertension	47 (92.2)
Diabetes mellitus	24 (47.1)
Dyslipidaemia	38 (74.5)
Current/heavy smoker (>20 pack-years)	13 (25.5)
Chronic obstructive pulmonary disease	7 (13.7)
Home oxygen therapy	1 (2.0)
Coronary artery disease / prior CABG / heart failure / arrhythmia	26 (51.0)
Peripheral artery disease / aortic aneurysm	30 (58.8)
Prior stroke / carotid surgery	9 (17.6)
Chronic kidney disease, stage 1-2 (GFR > 60 ml/min/1.73 m^2)	30 (58.8)
Chronic kidney disease, stage 3 (GFR 30-59 ml/min/1.73 m^2)	16 (31.4)
Chronic kidney disease, stage 4-5 (GFR ≤29 ml/min/1.73 m^2 or dialysis)	5 (9.8)
Kidney transplant recipient	1 (2.0)
Liver cirrhosis	1 (2.0)
Coagulopathy (Factor V Leiden; acquired haemophilia)	2 (3.9)
Myelodysplastic syndrome	1 (2.0)
Malignancy (≥ entity per patient)	14 (27.5)
Malignant melanoma	2 (14.3)
Non-small cell lung cancer	4 (28.6)
Renal cell carcinoma	2 (14.3)
Esophageal squamous cell carcinoma	1 (7.1)
Anal rim carcinoma	1 (7.1)
Breast cancer	1 (7.1)
Prostate cancer	3 (21.4)

Basal cell carcinoma	1 (7.1)
Chronic myeloid leukemia	1 (7.1)
Prior bowel resection due to ischaemia	2 (3.9)
Laboratory values at presentation	
Anaemia (Hb <13.5 g/dl, Hct <40%)	38 (74.5)
Glomerular filtration rate (MDRD) ml/min/1,73m^2	68.4 (38; 5-142)
Creatinine, mg/dl	1.5 (0.53; 0.46-7.66)
C-reactive protein, mg/dl	3.7 (2.8; 0.01-16.8)
Clinical presentation	
Unintended weight loss	40 (78.4)
Weight loss, kg/month	3.1 (3.75; 0.83-7.5)
Postprandial pain and food fear	46 (90.2)
(Bloody) diarrhoea	23 (45.1)
Obstipation	6 (11.8)
Endoscopically confirmed ischaemic gastritis/colitis	26 (51.0)
Mesenteric vascular status	
Chronic visceral artery disease distribution*	
Single-vessel disease	4 (7.8)
Double-vessel disease	15 (29.4)
Triple-vessel disease	32 (62.8)
Significant stenoses/occlusions on CT angiography	
Superior mesenteric artery – stenosis	36 (70.6)
Superior mesenteric artery – occlusion	11 (21.6)
Celiac artery – stenosis	29 (56.9)
Celiac artery – occlusion	14 (27.5)
Inferior mesenteric artery – stenosis	27 (52.9)
Inferior mesenteric artery – occlusion	13 (25.5)
Celiacomesenteric trunk – stenosis	3 (5.9)
Celiacomesenteric trunk – occlusion	0 (0)
Internal iliac artery status	
<70% bilateral stenosis	23 (45.1)
≥70% unilateral stenosis/occlusion and <70% stenosis contralateral	14 (27.5)
≥70% bilateral stenosis	5 (9.8)
≥70% unilateral stenosis + contralateral occlusion	3 (5.9)
Bilateral occlusion	6 (11.8)
Target lesion morphology (CTA and DSA)	
Plaque composition (CTA)	
Fibrous	1 (1.8)
Mixed (fibrous + calcified)	30 (52.6)
Calcified	27 (47.4)
Calcification severity (*Society of Vascular Surgery classification*)	
Mild (<25% circumference)	2 (3.5)
Moderate (25%-50% circumference)	12 (21.1)

Severe (>50% circumference)	43 (75.4)
Peripheral Academic Research Consortium (PARC) score (0-4)	
0 – No calcium	0 (0)
1 – Focal (<180° circumference (one side of vessel) and <1/2 of total lesion length)	2 (3.5)
2 – Mild (<180° circumference (one side of vessel) and ≥1/2 of total lesion length)	8 (14.0)
3 – Moderate (≥180° circumference (both sides of vessel at same location) and <1/2 of total lesion length)	18 (31.6)
4 – Severe (≥180° circumference (both sides of vessel at same location) and ≥1/2 of total lesion length)	29 (50.9)
Calcium morphology (CTA)	
Eccentric/nodular	34 (59.6)
Concentric/circumferential	23 (40.4)
Stenosis/occlusion level	
Proximal/ostial segment	51 (89.5)
Middle segment	6 (10.5)
Lesion length, mm	11.4 (9; 2-30)
Number of target vessel lesion lengths exceeding 15 mm	16 (28.1)
Chronic total occlusions (target vessel)	5 (8.8)

Data are presented as n (%) or median (IQR; range).

* Referring to the number of significantly affected visceral arteries - superior mesenteric artery, celiac artery, inferior mesenteric artery.

Abbreviations: SMA = superior mesenteric artery; CA = celiac artery; IMA = inferior mesenteric artery; IVL = intravascular lithotripsy; IQR = interquartile range; GFR = glomerular filtration rate; CTA = computed tomography angiography; DSA = digital subtraction angiography; Hb = hemoglobin; Hct = hematocrit; GFR = glomerular filtration rate; MDRD = modification of diet in renal disease; CABG = coronary artery bypass graft.

2) Procedure characteristics, IVL Performance, Adjunctive Therapy, Safety Outcomes, and Follow-up	
**Outcome/Variable**	**Value**
**Vascular access site**	
Common femoral artery	50 (98.0)
Brachial artery	1 (2.0)
**Revascularisation pattern per patient** (target and non-target vessels)	
Single-vessel: SMA	27 (52.9)
Single-vessel: CA	4 (7.8)
Single-vessel: IMA	2 (3.9)
Double-vessel: SMA + CA	14 (27.5)
Double-vessel: SMA + IMA	1 (2.0)
Double-vessel: CA + IMA	1 (2.0)
Triple-vessel: SMA + CA + IMA	2 (3.9)
**IVL target vessels**	
Superior mesenteric artery	38 (66.6)
Celiac artery	12 (21.1)
Inferior mesenteric artery	4 (7.0)
Celiacomesenteric trunk	3 (5.3)
**Number of IVL target vessels per patient**	
Single-vessel IVL	45 (88.2)
Double-vessel IVL	6 (11.8)
Additional non-target vessel revascularisation	11 (21.6)
**IVL target lesion characteristics and treatment results**	
**Baseline**	
Reference vessel diameter, mm – median (IQR; range)	6.2 (1.6; 2.7-9.5)
Minimum lumen diameter, mm – median (IQR; range)	1.7 (1.5; 0-4)
Diameter stenosis, % – median (IQR; range)	72.0 (23.3; 41-100)
Lesion length, mm – median (IQR; range)	11.4 (9; 2-30)
**IVL**	
Predilatation with plain angioplasty balloons	26 (45.6)
Successful IVL delivery across target lesion	57 (100)
IVL balloon rupture due to sharp calcium	2 (3.5)
Delivered IVL pulses per target lesion, pulses – median (IQR; range)	194.4 (20; 80-300)
**Post-IVL (prior to adjunctive therapy)**	
Residual diameter stenosis, % – median (IQR; range)	43.9 (13.8; 3.8-76)
Acute lumen gain, mm – median (IQR; range)	1.6 (1.3; 0-3.6)
Minimum lumen diameter, mm – median (IQR; range)	3.4 (1; 1.2-5.2)
**Adjunctive therapy**	

Plain balloon angioplasty	8 (14.0))
Drug-eluting balloon angioplasty	2 (3.5)
High-pressure balloon angioplasty	0 (0)
Atherectomy	0 (0)
Aspiration thrombectomy	2 (3.5)
Stent implantation (any)	53 (93.0)
Overlapping stent constructs	4 (7.0)
Bare-metal stents	47 (88.7)
0.014/0.018-inch delivery platform (Tsunami, Terumo; RX Herculink Elite Peripheral, Abbott; Palmaz Blue, Cordis)	9 (17.0)
0.035-inch delivery platform (BeSmooth, Bentley InnoMed; Visi-Pro, Medtronic)	38 (71.7)
Covered stents	6 (11.3)
0.014-inch delivery platform (PK Papyrus, Biotronik)	1 (1.9)
0.035-inch delivery platform (BeGraft, Bentley InnoMed)	5 (9.4)
**Final angiographic result**	
Minimum lumen diameter, mm – median (IQR; range)	5.3 (1.9; 1.7-9.5)
Maximum stent diameter, mm – median (IQR; range)	6.3 (1.5; 2.1-9.5)
Residual stenosis, % – median (IQR; range)	16.7 (11.7; 0-66)
Residual stenosis <30%/>50%	55/2 (96.5/3.5)
Relative stenosis reduction vs baseline, % – median (IQR; range)	76.0 (18.2; 21.3-100)
Acute lumen gain, mm – median (IQR; range)	3.5 (2.1; 0.5-7)
Mean stent expansion, % – median (IQR; range)	72.2 (18.9; 36.8-100)
**Technical success *** – n (%)	53 (93.0)
Procedure duration, min – median (IQR; range)	64.5 (36.3; 14-166)
Radiation exposure, mGy*cm² – median (IQR; range)	87642 (121656; 4049-342000)
Fluoroscopy time, min – median (IQR; range)	23.5 (14.2; 6.1-114)
Contrast volume, ml – median (IQR; range)	154 (88.8; 55-355)
Vascular closure device used	47 (92.2)
Clip-based	46 (97.9)
Suture-based	0 (0)
Collagen plug-based	1 (2.1)
Hospital stay, days – median (IQR; range)	5.7 (4; 2-23)
Discharge after the procedure, days – median (IQR; range)	3 (1; 1-17)
**Safety**	
Overall number of intra- and periprocedural adverse events	19 (37.3)
Intra-procedural AEs	
Mild AEs	
Self-limiting vasospasm	3 (5.9)
Non flow-limiting dissection (NHLBI A/B)	2 (3.9)
Moderate-to-severe AEs	
Flow-limiting dissection (NHLBI D)	3 (5.9)
Distal embolisation	2 (3.9)
Reperfusion haemorrhage (gastroduodenal artery)	1 (2.0)

Cardiac decompensation requiring intensive care	1 (2.0)
Early post-procedural AEs (POD 1-3)	
Access site complication requiring intervention (POD 1)	1 (2.0)
Acute stent thrombosis (POD 1)	1 (2.0)
Death (POD 1) – myocardial infarction	1 (2.0)
Mild reperfusion injury (POD 2-3)	3 (5.9)
Perforated cholecystitis with peritonitis (POD 3)	1 (2.0)
**Follow-up**	
Follow-up duration, days – median (IQR; range)	578.0 (529.5; 1-1696)
12-months follow-up rate	45 (91.8)
Patients lost to follow-up	2 (3.9)
**Clinical outcomes**	
Clinical symptom relief post-intervention	48 (98.0)
Pain reduction	44 (89.8)
Weight gain	25 (51.0)
Recovery from ischaemic gastritis/colitis	24 (92.3)
**Recurrence of chronic mesenteric ischaemia symptoms**	9 (18.4)
**Clinically-driven target-vessel revascularisation**	8 (16.3)
**Patency (clinical**), days – median (IQR; range)	
Primary patency	541.6 (415.5; 0-55)
Primary-assisted patency	299.8 (383.5; 1-546)
Secondary patency	185.0 (120; 58-298)
Asymptomatic in-stent restenosis on imaging	6 (12.3)
**Survival**	
Death during follow-up	8 (16.3)
Death related to acute-on-chronic mesenteric ischaemia	1 (2.0)

Data are presented as n (%) or median (IQR; range).

* Defined as successful IVL delivery with ≤30% residual stenosis after any adjunctive therapy.

Abbreviations: AEs = adverse events; SMA = superior mesenteric artery; CA = celiac artery; IMA = inferior mesenteric artery; IVL = intravascular lithotripsy; IQR = interquartile range; CTA = computed tomography angiography; DSA = digital subtraction angiography; NHLBI = National Heart, Lung, and Blood Institute; POD = post-procedural day.

3) Technical specifications of commonly used materials and devices	
**Material**	**Variant**
Sheaths/Introducer Systems	• Radifocus Introducer II (Terumo) – 5 Fr; 10 cm length<br>• Destination Guiding Sheath (Terumo) – 6 or 7 Fr; Multi-Purpose or Renal Double Curve; 45 cm or 90 cm length<br>• Flexor Check-Flo Introducer Set (Cook Medical) – 6 or 7 Fr; Ansel modification; 45 cm length
Guidewires	• Radifocus Guide Wire M Standard (Terumo) – 0.035-inch; angled 3 cm tip<br>• Radifocus Glidewire Advantage (Terumo) – 0.018-inch<br>• J Tip CHOICE PT Extra Support (Boston Scientific) – 0.014-inch<br>• Astato XS 20 (Asahi Intecc Medical) – 0.014-inch
Diagnostic/Support Catheters	• Beacon Tip Torcon NB Advantage Catheter (Cook Medical) – 5 Fr; curves C1/C2/VS2/VS3/SIM1/KMP/MPA; 80, 100, or 125 cm length<br>• Imager II (Boston Scientific) – 5 Fr; curves C1/C2; 80 cm length<br>• NaviCross Support Catheter (Terumo) – 4 Fr; 0.035-inch; 30° angled; 90 cm length<br>• Soft-Vu Pigtail (Angiodynamics) – 5 Fr
Intravascular Lithotripsy Balloon Catheters	• Shockwave C2/C2+ (Shockwave Medical) – OTW; diameter 4 mm, length 12 mm<br>• Shockwave S4 (Shockwave Medical) – OTW; diameter 4 mm, length 40 mm<br>• Shockwave M5/M5+ (Shockwave Medical) – OTW; diameters 4.5-7 mm, length 60 mm
Balloon Dilatation Catheters	• Armada 14 / Armada 18 (Abbott) – OTW; 0.014-/0.018-inch; diameters 2.5-5 mm, lengths 20-40 mm<br>• Pacific Plus PTA Catheter (Medtronic) – OTW; 0.018-inch; diameters 2.5-5 mm, lengths 20-40 mm<br>• Admiral Xtreme (Medtronic) – OTW; 0.035-inch; diameters 4-6 mm, lengths 20-40 mm<br>• InPact Admiral Drug-Coated Balloon (Medtronic) – OTW; 0.035-inch; diameter 5 mm, length 40 mm<br>• Selution SLR Drug-Eluting Balloon (Cordis) – OTW; 0.018-inch; diameters 4-6 mm, lengths 40-60 mm
Balloon-expandable Bare-Metal Stents	• RX Herculink Elite (Abbott) – Rx; 0.014-inch; cobalt-chromium; diameter 4 mm, length 15 mm<br>• Tsunami Peripheral (Terumo) – Rx; 0.018-inch; stainless steel; diameter 6 mm, lengths 12-18 mm<br>• Palmaz Blue (Cordis) – OTW; 0.018-inch; cobalt-chromium; diameters 5-6 mm, length 12 mm<br>• BeSmooth Peripheral (Bentley InnoMed) – OTW; 0.035-inch; cobalt-chromium; diameters 5-10 mm, lengths 18-38 mm<br>• Visi-Pro (Medtronic) – OTW; 0.035-inch; stainless steel; diameter 8 mm, length 17 mm
Balloon-expandable Covered Stents	• PK Papyrus (Biotronik) – Rx; 0.014-inch; cobalt-chromium; diameter 5 mm, length 26 mm<br>• BeGraft (Bentley InnoMed) – OTW; 0.035-inch; cobalt-chromium; diameters 5-6mm, lengths 18-38 mm

Abbreviations: OTW = over-the-wire; Rx = rapid exchange

4) Effect of Missing Follow-Up from Two Patients on Kaplan-Meier Estimates of Primary Patency and Survival at 6 and 12 Months:

Missing data were handled using three approaches: (1) imputation as “event occurred” (patency lost/death), (2) imputation as “no event” (patency maintained/alive), and (3) exclusion from the analysis as incomplete data sets. Results:

Kaplan-Meier survival analysis:

Handling of missing data	6-months survival	12-months survival
Imputed as deceased	88.2%	85.9%
Imputed as alive	91.8%	89.5%
Excluded	91.7%	89.4%

Kaplan-Meier primary clinical patency analysis:

Handling of missing data	6-months patency	12-months patency
Imputed as patency lost	89.7%	87.4%
Imputed as maintained patency	93.4%	91.0%
Excluded	93.4%	91.0%

Conclusion: Across approaches, the variation in estimated rates was modest (approximately 4%). Although missing data introduces bias, the sensitivity analysis demonstrated that the effect on the estimated patency and survival outcomes is limited.

5) Changes in Diameter Stenosis and Minimal Lumen Diameter at Baseline, after IVL and on the Final Angiogram:

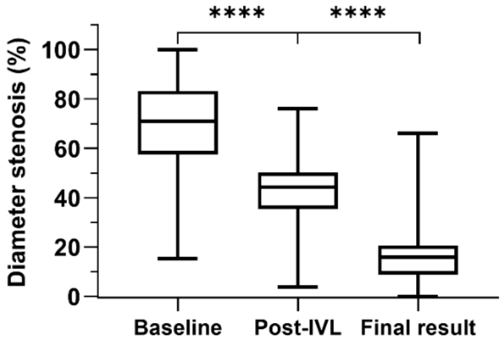

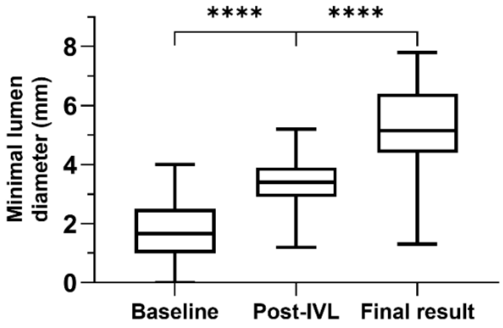

A mixed-effects analysis of the three matched/paired groups showed significant (****) changes after each measure (p<0.0001) underlining that IVL in the mesenteric vasculature should be used as a vessel preparation technique prior to mesenteric stenting and not as a stand-alone technique.
